# The Evaluation of Risk Factors for Vancomycin-Resistant Enterococcus Colonization and Infection Among Mixed Adult Intensive Care Unit Patients

**DOI:** 10.7759/cureus.33210

**Published:** 2023-01-01

**Authors:** Melek Doganci, Seval Izdes, Mustafa Ozgur Cirik

**Affiliations:** 1 Critical Care, Ankara Atatürk Sanatoryum Training and Research Hospital, Ankara, TUR; 2 Critical Care Medicine, Ankara Yıldırım Beyazıt University, School of Medicine, Ankara, TUR; 3 Anesthesiology and Reanimation, Ankara Atatürk Sanatoryum Training and Research Hospital, Ankara, TUR

**Keywords:** length of stay in icu, antimicrobial resistance infectious diseases, mdro colonization, medical intensive care unit (micu), vancomycin resistant enterococcus (vre)

## Abstract

Background and objective

Despite the adherence to strict infection control measures, vancomycin-resistant enterococcus (VRE) colonization and VRE infections are still important problems nowadays. However, there are only a limited number of studies examining the factors causing the transformation of VRE colonization to VRE infection in the intensive care unit (ICU).

The aim of this study is to delineate the prevalence of VRE colonization and its transformation into infection and the risk factors leading to infection.

Methods

Patients admitted to the third-level mixed-type ICU from 2012 to 2015 for at least 24 hours and acquired VRE colonization and VRE infection, both during and after their admission, were included in the study, and their medical records were examined retrospectively. VRE rectal swabs were taken weekly from each patient on admission and discharge from the ICU. If the VRE-positive patient was detected negative for VRE on the rectal swap taken three times in total as a surveillance culture successively, this patient was accepted as VRE negative. Demographic data, Acute Physiology and Chronic Health Evaluation II (APACHE-II) scores, invasive procedures, treatments (corticosteroid, antibiotic, etc.), nutrition types, laboratory results, and ICU results were recorded.

Results

Among 1730 patients admitted to ICU, 101 (5.8%) were found to carry VRE colonization. Twelve (11.8%) out of 101 patients colonized with VRE developed VRE infection. About 56.4% had urinary tract infections, 68.3% had pneumonia, 15.8% had surgical site infections, and 24.8% had catheter-associated infections among these infected patients. The most prevalent factor was *Enterococcus faecium *in patients with VRE colonization (64.3%) and infection (91%). VRE turned negative in 67% of patients with VRE colonization during their stay in ICU. Renal replacement therapy was statistically significant (p < 0.05) in the group with VRE infection (66.7%) compared to the VRE-colonized group (26.1%). Infection development risk among carriers of VRE for more than one week was again found statistically significant (p = 0.025). Demographic data, APACHE-II scores, treatments, nutrition type, previous antibiotic usage and types, invasive procedures, laboratory results, and ICU results were similar among the patients with VRE colonization and infection.

Conclusion

A longer duration of ICU stay in patients with colonization and previous renal replacement therapy increases the transformation of VRE colonization to VRE infection. Strategies toward decreasing VRE-colonized patients’ period of stay in ICU is the main objective to control the rate of VRE infection.

## Introduction

Enterococci present in the normal flora of the gastrointestinal system (GIS) of humans are related to endocarditis, wound infection, and urinary system infection and are also factors for nosocomial bacteremia. Despite the strict infection control measures taken, vancomycin-resistant enterococcus (VRE) colonization and VRE infections are still important problems nowadays. However, there are only a limited number of studies examining the factors causing the transformation of VRE colonization to VRE infection in the intensive care unit (ICU) [1]. Enterococci are microorganisms that could resist vancomycin, which causes colonization and infection in patients staying in hospitals. GIS is the major reservoir of *Enterococcus faecium*. Since people carrying VRE are usually asymptomatic, they could only be detected during surveillance studies. VRE colonization could persist for weeks, even months, especially in patients belonging to high-risk groups. Also, VREs might colonize in the flora of the female genital system other than GIS. Medical devices, tools, articles present in the patient room (bed, table, door knobs, electric switches, etc.), and surfaces are also important VRE sources. Devices commonly used on patients such as electronic thermometers and electrocardiogram electrodes could be responsible for the spread of VRE [2,3]. Since VRE strains exhibit multiple antibiotic resistance, they could become highly resistant to heat and disinfection; strains could continue their existence in a hospital medium for a considerable period of time [4].

For VRE colonization, patients who use oral or parenteral glycopeptide, metronidazole, cephalosporin, fluoroquinolone, carbapenem, beta-lactam antibiotics; patients who stay in hospital for long periods; patients having previous hospital infections; neutropenic patients; dialysis patients; organ transplant patients; central venous catheter patients; patients who are fed via an enteral tube; human immunodeficiency virus-positive patients; patients who had undergone intra-abdominal surgery, and patients having accompanying diseases are under risk [1,4-9]. Early identification of patients who are colonized with VRE and isolation measures prevent the spread of these microorganisms inside the hospital.

The number of patients infected or colonized with VRE could be decreased by determining and eliminating the risk factors for patients with VRE in the ICU. In Turkey, there are only a limited number of studies examining the risk factors of VRE colonization among patients in the ICU.

This study is aimed to retrospectively determine the prevalence of VRE colonization and infection at the time of ICU admission and during the stay in ICU as well as the risk factors causing these.

## Materials and methods

Patients admitted to mixed (surgery, medical, trauma)-type ICU with a capacity of 20 beds for at least 24 hours who were hospitalized from 2012 to 2015 at Yildirim Beyazit University Medical Faculty in Ankara Atatürk Training Research Hospital were included in the study. VRE rectal swab cultures were taken from each patient at their first admission to ICU. Medical records of patients with positive VRE colonization and who transformed to infection from colonization according to rectal swap cultures taken within the first 24 hours following the admission to the ICU and weekly rectal swap cultures taken weekly after admission were examined retrospectively from the hospital, ICU, and infectious diseases record system. If a patient’s result is negative for VRE according to the first swap taken within the 24 hours in ICU and on weekly rectal swaps taken three times in total consecutively, then the patient was accepted as negative for VRE. However, when VRE colonization/infection was identified in ICU, the screening cultures were continued in the unit weekly. If there are no other patients with VRE colonization and if the patients are negative for VRE, then VRE cultures were taken monthly until a patient is found positive for VRE.

In this study, information such as age; gender; the number of accompanying diseases; Acute Physiology and Chronic Health Evaluation II (APACHE-II) score within the first 24 hours; the length of time and history of stay in the hospice, hospital, or ICU within the last three months; the unit from which the patient was transferred to ICU or whether the patient was transferred from another hospital; the reason for admission to the ICU (surgery, medical, or trauma), and whether the patient was neutropenic were recorded. Factors such as whether the patient required hemodialysis, nutritional status (parenteral vs. enteral), enteral nutrition type (oral, nasogastric, percutaneous endoscopic gastrostomy, etc.), presence of nosocomial infection prior to VRE colonization, candidemia status, antibiotic usage, period and quantity prior to VRE colonization, steroid usage and quantity (prior to and/or after admission), whether the patient had postoperative (emergency/elective) and abdominal operation, and the date on which VRE was detected after admission or at the admission to VRE were detected. It was recorded whether the patient had a central venous catheter and/or a dialysis catheter, whether colostomy was performed prior to colonization, whether tracheostomy was performed or the patient was intubated, whether the patient was on invasive/noninvasive mechanical ventilators, and any other invasive operations (chest tube, angiography, bronchoscopy, etc.) were recorded. Albumin, creatinine, blood urea nitrogen (BUN), hemoglobin, alanine aminotransferase (ALT), and aspartate aminotransferase (AST) levels were recorded on the day when colonization was detected. Urinary tract infection, pneumonia, surgical site infection (skin-wound site infection), catheter infection, and other infection status were studied; if VRE infection was present, then its breeding site was detected and studied to determine whether it was bacteremia. The type of VRE colonized and/or causing infection in the patient, the period of carriage of patients colonized with VRE, the number of patients whose VRE colonization turned negative, and the period of ICU stay were recorded.

Statistical methods

Statistical analyses were evaluated with the SPSS 22.0 (Statistical Package for the Social Sciences, IBM Corp., Armonk, NY) package program. In the evaluation of data, frequencies and percentages were given for qualitative data. Kolmogorov-Smirnov test was used to determine the normal distribution of quantitative data. Mean and standard deviation values from descriptive statistical methods were provided for quantitative data, and frequency and percentage (%) values were provided for qualitative data.

## Results

Among 1730 patients admitted to ICU, 101 (5.8%) were found to carry VRE colonization. VRE infection developed in 11 (10.9%) of the patients with VRE colonization. A total of 42 male and 59 female patients were studied, and the average age of the patients was 75 ± 17.8. Considering the underlying diseases of 91.1% of patients, 41.6% had heart disease, 51.5% had hypertension, 32.7% had diabetes mellitus (DM), 19.8% had kidney failure, 17.8% had asthma, 5% had hematologic malignancy, and 5% had liver disease. The average APACHE-II score of patients was 25 ± 6.2; the lowest APACHE-II score was 7, and the highest APACHE-II score was 38. The average hospital stay of patients was 42 ± 64.2 days, and their ICU stay was 37 ± 62.9 days. VRE colonization was detected on the 20 ± 29th day of their admission to the hospital and on the 13 ± 27.7th day of their admission to the ICU; 18.8% of patients came to our hospital from an external center or hospice. The majority of patients admitted to ICU were patients from the emergency service with a ratio of 39.6% and patients coming from the orthopedics with a ratio of 17.8%. About 12.9% of patients were admitted to ICU after a postoperative emergency operation, and 13.9% of patients were admitted to ICU after a postoperative elective operation. There was at least one dose of steroid usage history in 42.6% of patients in the ICU from admission to the hospital to the detection of VRE. About 16.8% of patients had catheter-mediated parenteral nutrition, 80.2% of patients had enteral nutrition, and the majority of enteral nutrition patients were treated via nasogastric feeding with a ratio of 55.4%. About 29.7% of patients had at least one hemodialysis therapy, tracheostomy was applied to 14.9%, and intubation was applied to 57.4% of patients until VRE was detected from the date of admission to the hospital. About 83.2% of patients had a central catheter, and 6.9% had a colostomy. When it was examined to see whether other infections are present, 56.4% of the patients had urinary tract infections, 68.3% had pneumonia, 15.8% had surgical site infections, and 24.8% had catheter-associated infections. About 81.2% of the patients had a focus of infection prior to VRE, and 81.2% had a focus of infection prior to VRE. During their stay in the ICU, 67.3% of the patients with VRE colonization became VRE negative. It was found that 19.8% of hospital admissions were due to surgery, 70.3% were due to medical reasons, and 7.9% were due to trauma. Invasive procedures were not applied to 76.8% of patients, bronchoscopy was applied to 5.9%, the chest tube was applied to 11.9%, angiography was applied to 4%, and colonoscopy was applied to 1% of patients. About 96% of the patients did not have neutropenia. The most prevalent factor was *E. faecium* in patients with VRE colonization (27.7%) and infection (91%) (Table 1).

**Table 1:** Demographic and other features of patients Mean ± standard deviation (minimum-maximum values) or numbers (%). VRE: Vancomycin-resistant enterococcus, APACHE: Acute Physiology and Chronic Health Evaluation, PEG: Percutaneous endoscopic gastrostomy, ICU: Intensive care unit.

Parameters	Having VRE colonization (n = 101) (%)
Age (years)	75 ± 17.8 (18-101)
Gender (F/M)	59 (58.4)/42 (41.6)
Having accompanying diseases	92 (91.1)
Heart disease	42 (41.6)
Hypertension	52 (51.5)
Diabetes mellitus	33 (32.7)
Kidney failure	20 (19.8)
Asthma	18 (17.8)
Hematological malignancy	5 (5)
APACHE-II score	25 ± 6.2 (7-38)
From which unit of the hospital	
Emergency	40 (39.6)
Brain surgery	3 (3)
General surgery	3 (3)
Internal medicine	11 (10.9)
Infectious diseases	5 (5)
Chest diseases	6 (6)
Hematology	1 (1)
Obstetrics and Gynecology	1 (1)
Cardiology	3 (3)
Neurology	5 (5)
Orthopedics	18 (17.8)
Plastic surgery	3 (3)
Urology	2 (2)
Causes of hospitalization	
Medical	71 (70.3)
Surgical	20 (19.8)
Trauma	8 (7.9)
Postoperative (emergency) patients	13 (12.9)
Postoperative (elective) patients	14 (13.9)
Patients using steroids	43 (42.6)
Parenteral nutrition	17 (16.8)
Enteral nutrition	81 (80.2)
Nasogastric	56 (55.4)
PEG	9 (8.9)
Oral	16 (15.8)
Need for hemodialysis	30 (29.7)
Number of patients having tracheostomy	15 (14.9)
Number of patients having intubation	58 (57.4)
Current infections	
Urinary tract infection	57 (56.4)
Pneumonia	69 (68.3)
Surgical site infection	16 (15.8)
Catheter infection	25 (24.8)
Other infections	8 (7.9)
Having VRE infection	11 (10.9)
VRE species	
Faecalis	13 (12.9)
Faecium	28 (27.7)
Gallinarum	2 (2)
Cloaca	2 (2)
Aerogenes	1 (1)
Not specified	10 (9.9)
Carriage time less than 1 week/Longer than 1 week	54 (53.5)/45 (44.6)
VRE turned negative	68 (67.3)
Number of patients using antibiotics before colonization	82 (81.2)
Neutropenic patients	4 (4)
Invasive procedures	
Bronchoscopy	6 (5.9)
Chest tube	12 (11.9)
Angiography	4 (4)
Colonoscopy	1 (1)
Patients with a central catheter	84 (83.2)
Patients with colostomy	7 (6.9)
External center/hospice stay	19 (18.8)
Mortality (28 days)	52 (51.8)
Length of hospital stay (day)	42 ± 64.2 (3-370)
Length of stay in ICU (day)	37 ± 62.9 (2-365)

According to the blood work carried out at the date of detection of VRE colonization, the blood value average of all patients was albumin: 2.8 ± 0.45, creatinine: 1.1 ± 1.23, BUN: 73.5 ± 57.8, aspartate transaminase (AST): 29.5 ± 346, alanine transaminase (ALT): 22.5 ± 258, and hemoglobin: 8.25 ± 2.9 (Table 2).

**Table 2:** The assessment of laboratory values of cases in the group Mean ± standard deviation (minimum-maximum values). VRE: Vancomycin-resistant enterococcus; BUN: Blood urea nitrogen; ALT: Alanine transaminase; AST: Aspartate transaminase.

Parameters	Having VRE colonization (n = 90) (min-max)	Having VRE infection (n = 11) (min-max)	Our reference range	P-values
Hemoglobin (g/dL)	8.35 ± 3.09 (5.4-12.6)	8.20 ± 0.88 (7.1-9.6)	11.7-17.5	0.604
BUN (mg/dL)	67 ± 57.15 (8-214)	103 ± 61.71 (52-250)	17-43	0.081
Creatinine (mg/dL)	1.1 ± 1.24 (0.1-6.6)	1.8 ± 1.19 (0.35-4.2)	0.51-1.09	0.185
Albumin (g/dL)	2.8 ± 0.45 (1.8-4.3)	2.71 ± 0.46 (1.7-3.3)	35-52	0.772
ALT (U/L)	21.5 ± 274.7 (1-2538)	24.0 ± 17.08 (4-54)	1-35	0.559
AST (U/L)	29 ± 368.3 (8-3472)	35 ± 61.5 (13-232)	1-35	0.525

When the patients having VRE colonization and VRE infection were compared via chi-square test to determine the risk factors, patients having renal replacement therapy were found statistically significant (p = 0.017). Also, infection development risk among carriers of VRE for more than one week was again found statistically significant (p = 0.025) (Table 3).

**Table 3:** The assessment of cases in the groups according to their demographic and other features Mean ± standard deviation (minimum-maximum values) or numbers (%). VRE: Vancomycin-resistant enterococcus; MV: Mechanical ventilation.

Parameters	Subparameters	Having VRE colonization (n = 90) (%)	Having VRE infection (n = 11) (%)	P-value
Age (years)		67.16 ± 18.1 (20-100)	70 ± 17.8 (18-101)	0.208
Having accompanying diseases		81 (90)	11 (100)	0.592
Number of patients having tracheostomy		14 (15.5)	1 (9.1)	1
Number of patients having intubation		52 (57.7)	6 (54.5)	1
Number of patients who apply MV		62 (68.8)	7 (63.6)	0.731
Current infections	Urinary tract infection	49 (54.4)	8 (72.7)	0.346
	Pneumonia	62 (68.8)	7 (63.6)	0.731
	Surgical site infection	15 (16.6)	1 (9.1)	0.687
	Catheter infection	20 (22.2)	5 (45.4)	0.139
	Other infections	6 (6.6)	2 (18.1)	0.217
Number of patients using antibiotics before colonization		71 (78.8)	11 (100)	0.203
Causes of hospitalization	Medical	61 (67.7)	10 (90.9)	
	Surgical	19 (21.1)	1 (9.1)	
	Trauma	8 (8.8)	0 (0)	
External center/hospice stay		18 (20)	1 (9.1)	0.685
Postoperative (emergency) patients		12 (13.3)	1 (9.1)	1
Postoperative (elective) patients		14 (15.5)	0 (0)	0.35
Patients using steroids		41 (45.5)	2 (18.1)	0.107
Neutropenic patients		4 (4.4)	0 (0)	1
Patients with candidemia		26 (28.8)	3 (27.2)	1
Patients with central catheter		73 (81.8)	11 (100)	0.207
Patients with colostomy		6 (6.6)	1 (9.1)	0.574
Parenteral nutrition		15 (16.6)	2 (18.1)	1
Enteral nutrition		73 (81.1)	8 (72.7)	0.437
Need for hemodialysis		23 (25.5)	7 (63.6)	0.017
Mortality (28 days)		44 (48)	7 (63.6)	0.527
Carriers longer than 1 week		36 (40)	9 (81.8)	0.025
Length of stay in ICU (day)		41.5 ± 65.5 (2-365)	57 ± 43.2 (7-141)	0.776
Length of hospital stay (day)		19.5 ± 66.8 (3-370)	32 ± 45.1 (8-145)	0.533

## Discussion

The aim of this study is to delineate the prevalence of VRE colonization and its transformation into infection and the risk factors leading to infection. If we know the risk factors, we can reduce the rate of VRE infection by taking measures to prevent them. In a meta-analysis of 37 studies, the rates of VRE infection among colonized patients ranged from 0% to 45% [10]. In our study, we found the rate of VRE infection in colonized patients as 10.9%, which is similar to other studies.

A total of 42 male and 59 female patients having VRE colonization/infection were included in this study. Considering the underlying diseases of 91.1% of patients, 41.6% had heart disease, 51.5% had hypertension, 32.7% had DM, 19.8% had kidney failure, 17.8% had asthma, 5% had hematologic malignancy, and 5% had liver disease. In a study demonstrating the increase of VRE risk by DM, it is specified that both hyperglycemia and corticosteroid usage are causing an additional immune system dysfunction by decreasing the clearance of organisms entering into the bloodstream from the gastrointestinal system. Also, corticosteroid usage might decrease the integrity of the gastrointestinal system and skin [11]. In our study, there was at least one dose of steroid usage history in 42.6% of patients in ICU from admission to the hospital to the detection of VRE as well. However, when two groups having VRE colonization and VRE infection were compared, a significant difference was not found in terms of DM and corticosteroid usage. In a retrospective, single-center study covering 53 patients with VRE colonization, neutropenic patients commonly diagnosed with acute myeloid leukemia and hematopoietic stem cell transplantation were studied, and approximately 38% of patients developed a VRE infection [12]. In our study, 5% of patients had hematologic malignancy, and 4% of complete patients had neutropenia. Although we could not find a correlation between VRE and neutropenia and accompanying diseases, it is commonly known that multiple comorbidities increase the ICU stay. A longer ICU stay increases the possibility of taking antibiotics and possible pathogen infection risk as well. For this reason, it is very important to take infection control measures. In a study involving pediatric oncological patients, the duration of neutropenia, the number of antibiotic agents, and the duration of antibiotic treatment were shown as risk factors, and it was found that environmental contamination had a significant effect on the patient-to-patient transmission of VRE and interventions that included the application of infection control measures were associated with a decrease in the incidence of gastrointestinal colonization [13].

As longer staying periods in the ICU increases the possibility of receiving a longer antimicrobial treatment, the risk of infections resistant to antibiotics may also increase. VRE incidence could be reduced by decreasing the length of stay in the ICU and vancomycin usage [14]. Newly acquired VRE incidence during ICU stay was examined in a study, in which 871 patients were screened, and length of stay in the ICU was found as an independent risk factor [15]. Patients having colonization with VRE were evaluated at two ICUs in a teaching hospital in Sao Paulo, and it was found that the only risk factor significantly associated with VRE under intensive-care conditions was the length of stay in the ICU [16]. In our study, we found that the risk of developing infection increased in colonized patients who were carriers for more than one week. Strategies to reduce the length of stay in the ICU of patients who remain colonized in the ICU for more than one week will also enable us to control the rate of VRE infection. In addition, the length of stay in the hospital before admission to the ICU was also found to be an independent risk factor for VRE carriage [10,14]. In our study, when the mean hospital stay of colonized patients and patients with VRE infection were compared, no significant change was observed.

A prospective, observational study in Brazil found that antibiotic use and carbapenem use prior to VRE were independent risk factors for VRE colonization [17]. In a study involving 43 ICUs in Italy, antibiotics were used in 75% of the patients in the ICU without sepsis, and no cause could be identified in 20% of them, and the majority was called "prophylaxis." It was emphasized that the unnecessary use of antibiotics is very common [18]. The American Thoracic Society Guideline recommends the use of early and appropriate antibiotics to reduce nosocomial infections. In our study, there were 82 patients who had infections before VRE and were using antibiotics, but no significant difference was observed between the two groups when the groups were compared.

In a study, 24 renal patients with VRE colonization/infection were compared with 29 renal patients with vancomycin-susceptible enterococcal infection, and a greater VRE risk was detected in patients with severe renal disease [19]. In another study, it was indicated that hemodialysis was a significant risk factor for the acquisition of VRE colonization/infection in all the patients hospitalized; however, it was also indicated that end-stage renal failure was not a risk factor [20]. It is known that central venous catheters and dialysis catheters applied before hemodialysis can also be a source of infection [21]. For this reason, the association between VRE and central catheters was searched in various studies. In a study carried out by Sakka et al., invasive devices were found as a risk factor for VRE. However, it is not evident whether the catheters are functioning as a primary channel for VRE infection or whether only weakness, long hospitalization, and more frequent catheter usage in patients having serious comorbidity are causing the infection [22]. Similarly, in our study, statistically significant results were obtained when renal replacement therapy is compared with the group with VRE infection (63.6%) and the group with VRE colonization (25.5%) but there was no correlation between invasive operations and central catheter with VRE infection development. Comorbidities of hemodialysis patients, their frequent exposure to other bacterial factors, and immune system deficiency are in parallel with these factors, and as patients having longer hemodialysis treatment stay in the hospital for a longer period of time, we believe that hemodialysis is a risk factor for VRE.

The retrospective nature of our study brought some limitations. Patients coming from an external center or hospice constituted 18.8% of all patients. As a result, it was not possible to determine whether these patients had previous colonization/infection and, if so, how long they had been colonized or infected. This could have had an impact on our statistical data.

## Conclusions

In this study, we determined that patients receiving hemodialysis treatment and patients with long-term VRE carriers are at a high risk of VRE infection. Consequently, early detection of patients with VRE colonization is vital to prevent VRE infection and to take measures against VRE infection. Logically, in order to decrease the VRE infection rate, our main target has to be the early detection of patients with VRE colonization and the implementation of strategies that are intended for decreasing the period of stay in the ICU.
